# A thermal deformation optimization method for cryogenically cooled silicon crystal monochromators under high heat load

**DOI:** 10.1107/S1600577523010664

**Published:** 2024-01-22

**Authors:** Jiayin Liu, Zhan Ji, Yichen Fan, Xinxin Yan, Miaomiao Wang, Hongliang Qin

**Affiliations:** a Synchrotron Radiation Facility, Institute of Advanced Science Facilities, Guangming, Shenzhen, Guangdong 518107, People’s Republic of China; Australian Synchrotron, Australia

**Keywords:** monochromator, silicon crystal, thermal deformation, cryo-cooled scheme

## Abstract

A method to optimize the thermal deformation of an indirectly cryo-cooled silicon crystal monochromator exposed to intense X-rays at a low-emittance diffraction-limited synchrotron radiation source is presented. The thermal-induced slope error of the monochromator crystal has been studied as a function of heat transfer efficiency, crystal temperature distribution and beam footprint size. A partial cooling method is proposed, which flattens the crystal surface profile within the beam footprint by modifying the cooling contact area to optimize the crystal peak temperature. The optimal temperature varies with different photon energies, which is investigated, and a proper cooling strategy is obtained to fulfil the thermal distortion requirements over the entire photon energy range. At an absorbed power up to 300 W with a maximum power density of 44.8 W mm^−2^ normal incidence beam from an in-vacuum undulator, the crystal thermal distortion does not exceed 0.3 µrad at 8.33 keV. This method will provide references for the monochromator design on diffraction-limited synchrotron radiation or free-electron laser light sources.

## Abstract 2

A partial cooling method is proposed for minimizing thermal deformation of the monochromator crystal at low-emittance diffraction-limited synchrotron radiation beamlines. The influence of the heat transfer efficiency, crystal temperature distribution and beam size on crystal surface was investigated, and a set of thermal deformation optimization techniques for high-heat-load monochromators was developed.

## 1. Introduction

With the advent of high-repetition-rate free-electron lasers (FELs) and diffraction-limited storage rings (DLSRs) based on multi-bend achromat (MBA) designs, the requirement to deliver and preserve brighter and more coherent X-ray beams is becoming increasingly stringent on optics surface figure and slope accuracy (Eberhardt, 2015 ▸; Eriksson *et al.*, 2014 ▸; Susini *et al.*, 2014 ▸; Zhang *et al.*, 2023 ▸). Thermal deformation caused by the intense X-ray beam poses an important issue for beamline optical elements. For hard X-ray monochromators, the crystal thermal deformation would not only broaden the rocking curve, resulting in flux loss and worse energy resolution, but also deform the wavefront, which has a negative impact on beam angular divergence and coherence preservation. In order to remove the high heat loads efficiently and improve optical performance, several liquid nitro­gen (LN2) cooled silicon crystal monochromators have been applied successfully at high heat loads since the early 1990s (Lee *et al.*, 1995 ▸; Mochizuki *et al.*, 1995 ▸; Shastri *et al.*, 2002 ▸; Wang *et al.*, 2010 ▸; Stimson *et al.*, 2019 ▸), owing to the combined advantages of high thermal conductivity and low thermal expansion coefficient at cryogenic temperatures for silicon crystal (Zhang, 1993 ▸; Lee *et al.*, 2000 ▸, 2001 ▸). Subsequently, extensive research has been conducted successively on high-heat-load monochromator cooling techniques (Cao *et al.*, 2011 ▸; Khorunzhii *et al.*, 2003 ▸).

The fact that a local minimum of crystal thermal deformation within the beam footprint is obtained when the peak temperature is near 165 K (the temperature of zero thermal strain, relative to *T*_LN2_) rather than 125 K (*T*_zero_, the temperature of zero instantaneous coefficient of thermal expansion for Si crystal), as the thermal strain is the product of the secant coefficient of thermal expansion α^se^ and temperature change, is highlighted and widely accepted (Zhang *et al.*, 2013 ▸). The nonlinear thermal deformation of Si crystal with temperature was studied by first-principles methods, and verified by finite-element analysis (FEA) in conjunction with experimental measurements on I16 beamline at Diamond Light Source (Khosroabadi *et al.*, 2022 ▸). As in this literature, it has been explained that the shape and amplitude of crystal thermal deformation are determined by the characteristic temperatures (*T*_b_, crystal body temperature; *T*_p_, surface peak temperature) of a given crystal, varying with different heat power distribution and cooling configuration. Hence an important conclusion was drawn that the optimal profile would be obtained when *T*_p_ > *T*_zero_ > *T*_b_. Furthermore, according to the thermal deformation equations [refer to equations (5) and (6) of Khosroabadi *et al.* (2022 ▸)] and the integration principle, it is easy to deduce that the optimal peak temperature *T*_p_ should be controlled between 125 K and 165 K, which will vary depending on the total absorbed power, power density, footprint size and cooling conditions. Consequently, the monochromator crystal thermal profile can achieve an almost perfect shape by optimizing the characteristic temperatures (*T*_p_ and *T*_b_) accordingly, even under varying heat loads as long as it does not exceed an extremely high limit, the start of the nonlinear region (Zhang *et al.*, 2003 ▸). The thermal conduction effect is strongly associated with the multiplication of the thermal contact conductance and the Cu–In–Si cooling interface contact area (*k* × *A*). In this paper, a partial cooling method (PCM) for thermal deformation optimization of indirectly LN2-cooled monochromators is proposed based on this idea.

An ideal shape of the monochromator crystal surface with sub-µrad accuracy is essential for high-performance X-ray beam delivery under intense heat power from advanced undulators on DLSRs. However, this is more and more difficult to realize with the present cooling schemes, especially under a high power density of tens of W mm^−2^. In this paper, a PCM for indirectly LN2-cooled Si monochromators is presented. The correlation between crystal peak temperature, the optical profile and the cooling contact area is calculated and explored by FEA, and the results are verified by ray-tracing simulations systematically. The method is validated on a cooling model, which can meet the requirements of sub-µrad accuracy with a high peak power density of 44.8 W mm^−2^ normal incidence beam, over a wide photon energy range.

## 2. Finite-element modelling

### 2.1. Heat load

**Figure 1:**
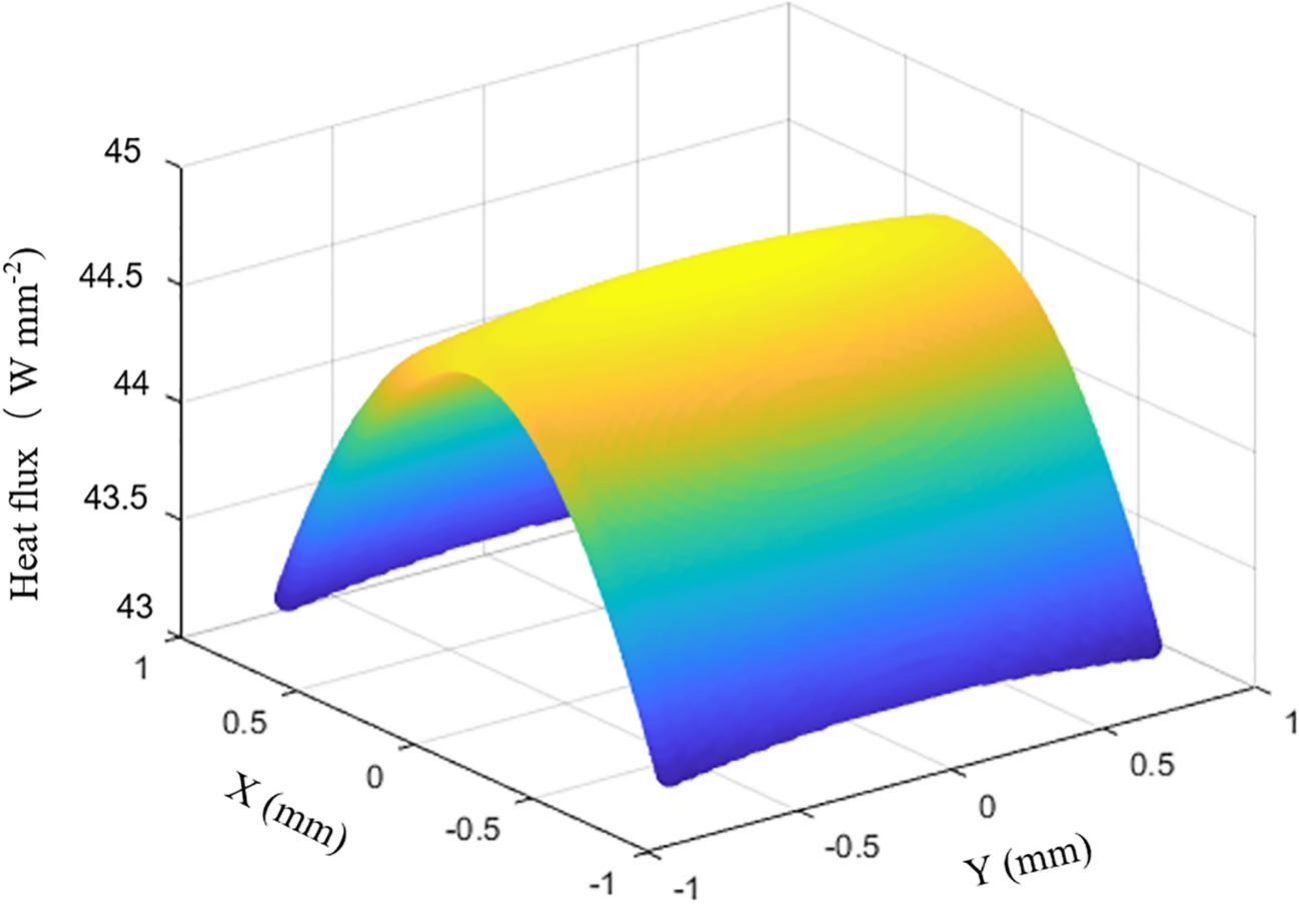
Heat power distribution on the monochromator, which is located at a distance of 36 m from an IVU22 light source.

An LN2-cooled monochromator in an in-vacuum undulator (IVU) beamline is taken as an example for optimization and validation in this study. The beamline is equipped with a 4.5 m-long 22 mm-period undulator and a minimum magnetic gap of 6 mm. The polychromatic power in the central cone is 110 W at a current of 300 mA. The monochromator is placed at 36 m from the light source, with one pair of Si (111) crystals, in which the photon energy of the reflected radiation can be tuned from 2.05 to 16 keV. The normal-incidence beam size at the monochromator is 1.62 mm × 1.62 mm (H × V). Here and below, all values of the beam size and angular divergence are given in the form of horizontal × vertical. The heat power density distribution in the plane perpendicular to the incidence beam at the monochromator is shown in Fig. 1 ▸, having a total power of 110 W with a maximum power density of 44.8 W mm^−2^. The heat power is calculated using the *SPECTRA* code (Tanaka & Kitamura, 2001 ▸). Herein, we assume that the illuminated heat power is absorbed by the crystal surface. In practice, the total absorbed power is 10–14% smaller than the calculated one due to beam scattering on the crystal surface (Zhang *et al.*, 2013 ▸).

### 2.2. Material properties

Due to the high heat load, the crystal temperature can change over a large range. The coefficient of thermal expansion (CTE) and thermal conductivity are nonlinear with temperature [refer to Zhang *et al.* (2013 ▸)], which has been reported previously and applied widely. Thus, parameters dependent on temperature are adopted. The thermal-mechanical properties utilized in the following simulations are listed in Table 1 ▸.

### 2.3. Finite-element model

**Figure 2:**
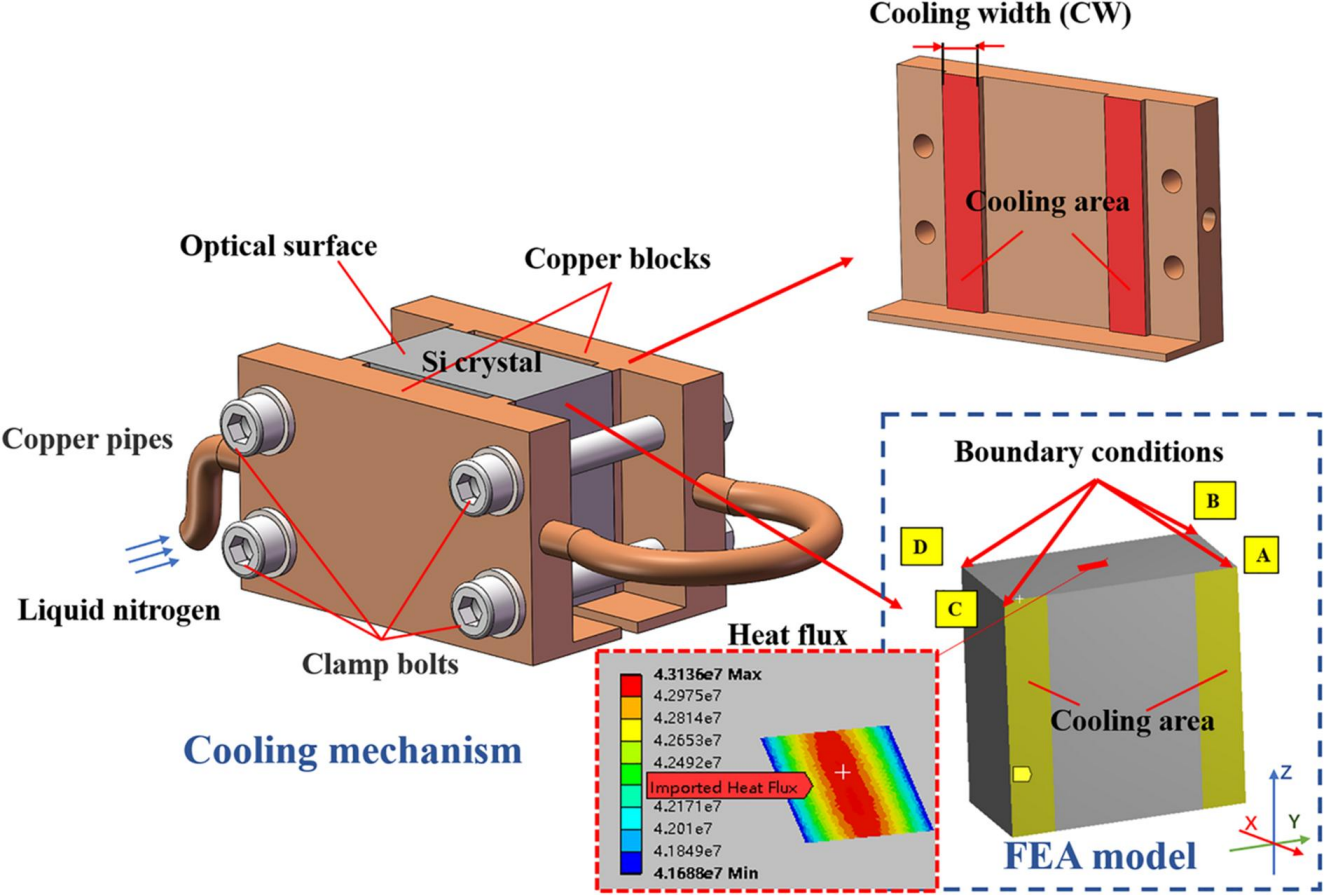
Schematic diagram of the cooling model and boundary conditions. The cooling contact area (yellow rectangular areas on crystal side surfaces) between the copper blocks and the crystal is adjustable with cooling width.

The finite-element model of the monochromator crystal cooling scheme is shown in Fig. 2 ▸. The crystal, whose dimensions are 60 mm (length) × 30 mm (width) × 60 mm (height), is indirectly cooled by LN2 and sandwiched with 0.5 mm-thick indium foils between two copper blocks (heat absorbers). Here a novel optimization method is presented by varying the interface contact area between the copper blocks and the crystal, which is called the partial cooling method. The optimal thermal slope error of the crystal can be achieved by modifying the cooling width (CW) for a given heat power distribution. In order to simplify the calculation, the crystal cooling model is replaced by the boundary conditions in the form of environmental heat convection: cooling contact zones (two yellow zones on each side, as shown in Fig. 2 ▸) are taken as heat exchanging areas, and the ambient temperature is set to *T*_LN2_ = 77 K. The cooling coefficient of the Cu–In–Si interface principally depends on the surface quality of the contact bodies and the contact pressure. Usually, the crystal and copper block cooling surfaces are polished in order to decrease the contact thermal resistance, between which the added indium foils are to improve thermal contact. The effective convection cooling coefficient of 3000 W m^−2^ K^−1^ is applied to the crystal side cooling surfaces in this case. This can be achieved by a clamping force of 5 bar. The influence of this clamping force on the crystal shape error is evaluated by FEA without regard to the interfacial friction. The clamping-induced slope error is about 0.1 µrad RMS, which is a negligible fraction of the overall slope in the form of convolution compared with the thermal deformation. In addition, it could be further diminished by modifying the clamping mechanism design. In this study, we mainly focus on the optimization of crystal thermal deformation, ignoring the mechanical-induced deformation.

As shown in Fig. 2 ▸, mechanical boundary conditions impose constraints on the structure in a statically determined way and hold it constraint-free regarding following thermal strain: a simply supported boundary condition is applied on the model. Point A is fixed on the three orthogonal translational degrees of freedom (*XYZ* directions). Limit the *YZ* and *XZ* translation freedoms for the two points adjacent to A, *i.e.* points B and C, respectively. It is fixed only in the *Z* direction for point D. Thus, the overall drift and tilt caused by disturbance and calculation error can be avoided, while the space for expansion and contraction are reserved sufficiently.

## 3. Simulation and discussion

### 3.1. Thermal deformation and peak temperature

A series of simulations are carried out based on the crystal model above. The thermal deformation displacement normal to the diffraction surface along the footprint length (in the *Y* direction) is calculated. The slope error in the meridional direction is obtained from the derivative of the normal displacement to the coordinate *Y*, which is very sensitive to the flux and used to evaluate the thermal deformation. Note that the minimum RMS thermal slope error is obtained by modifying the CW at the given heat load described in Section 2.1. Considering the worst case of the heat load effect, the Bragg angle of the incident beam is 74.7°, *i.e.* the maximum projected power density situation, corresponding to an equivalent photon energy of 2.05 keV. The CW ranges from 6 mm to 30 mm.

**Figure 3:**
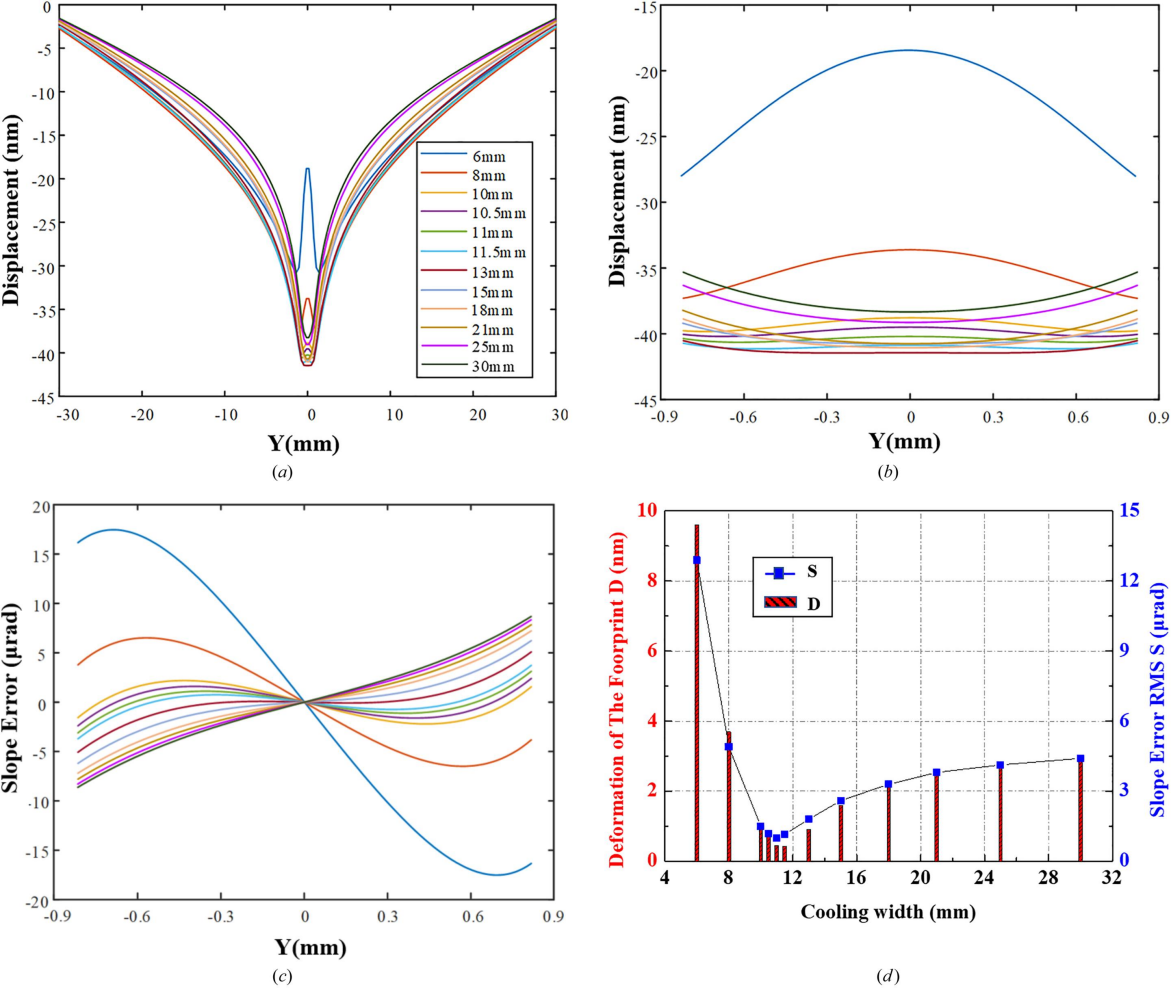
Results of thermal deformation for various cooling widths (from 6 mm to 30 mm). Displacement along the crystal full-length (*a*) and within the footprint (*b*). (*c*) Slope error within the footprint. (*d*) Crystal thermal deformation and slope error within the footprint with decreasing CW, showing a slow descent and rapid ascent trend.

The evolution of thermal deformation with CW is plotted in Fig. 3 ▸. It can be observed in Figs. 3 ▸(*a*) and 3 ▸(*b*) that there is a significant overall concave deformation trend outside the footprint area, which is due to the much lower temperature from the margin of the footprint to the crystal surface edge (it will converge rapidly towards *T*_LN2_ below 125 K). The CTE of monocrystalline silicon is negative between 77 K (*T*_LN2_) and 125 K, and its absolute value increases with decreasing temperature. Therefore, although the external temperature gradient around the illuminated area clearly drops, significant deformation accumulation is generated under the combined effect of the continuously increasing CTE absolute value towards the edges of the crystal; while in some cases of narrower CW a convex rebound is observed within the footprint area. The crystal surface deformation undergoes three stages with decreasing CW. The crystal is much deformed in a concave shape when CW is 30 mm, *i.e.* the whole crystal side surfaces are both cooled, which is identical to the mostly used cooling scheme in cryo-cooled monochromators. When CW varies from 30 mm towards 13 mm, the thermal contraction displacement increases gradually, whereas the concave deformation in the illuminated area exhibits a slight decrease. Then, the thermal contraction gradually recovers and the footprint profile flattens with the CW narrowing until ∼11 mm. As CW continues to narrow, the central crystal deforms from concave to convex shape. Finally, the central crystal deforms dramatically to a convex shape with a CW of 6 mm. The thermal slope error variation can be clearly seen in Figs. 3 ▸(*c*) and 3 ▸(*d*). The varying trend of the thermal slope error is similar to the deformation (peak to valley of the footprint area) and is characterized by a slow descent and rapid ascent with decreasing CW. There is a minimum slope error corresponding to the optimal cooling width, where the crystal deformation is in the process of converting from concave to convex, with minimal deformation. The optimal thermal slope error of 1 µrad RMS is obtained when CW is 11 mm in this case. Consequently, when regarding to 2.05 keV, the optimized CW reduces the thermal slope error by 77% compared with the conventional cooling scheme (CW = 30 mm). The found minimum value improvement is true for any given situation (heat load, beam size, *etc*.).

**Figure 4:**
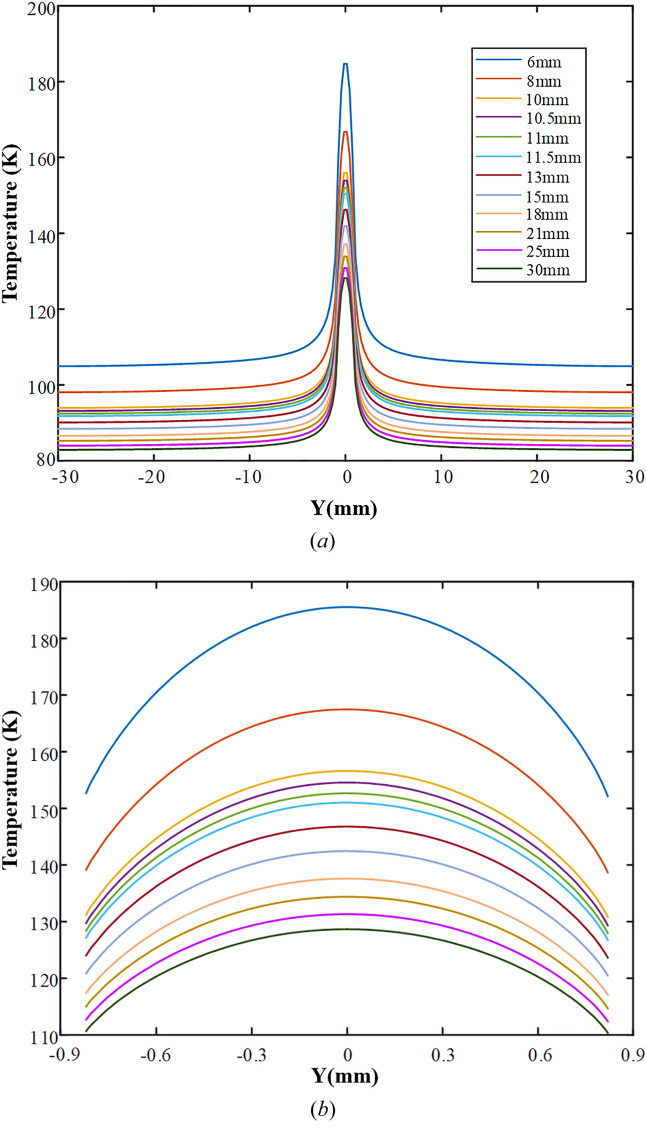
Crystal temperature distribution (along the beam footprint length) versus cooling width.

Fig. 4 ▸ shows the crystal temperature variations along the beam footprint length with the CW. In general, the cooling efficiency decreases with the narrowing of the CW, whereas the crystal peak temperature and the maximum absolute/relative temperature-rise increase accordingly. Herein the peak temperature of the crystal increases from 128.7 K to 185.5 K as CW decreases from 30 mm to 6 mm. When CW is in the interval 10.5–11.5 mm, the average peak temperature is ∼153 K and the thermal slope error can be controlled to less than 1.2 µrad. Specifically, the minimum thermal slope error (1 µrad) is achieved at the peak temperature of 152.6 K, just within the optimal temperature range predicted above, which confirms the correctness of the partial cooling theory.

The above cooling model can be designed and optimized for any specific heat loads in order to achieve sufficiently high shape accuracy. It is optimized to a minimum slope error at the given heat load regarding the maximum Bragg angle situation in the above case. Generally, the intrinsic rocking curve width of a low-energy beam far exceeds that of a high-energy beam; therefore it is unreasonable for a monochromator to demand the same slope error level over a wide photon energy range. It is essential to set a high accuracy for higher photon energy and a moderate one for lower photon energy.

### 3.2. Verification over the entire photon energy range

**Figure 5:**
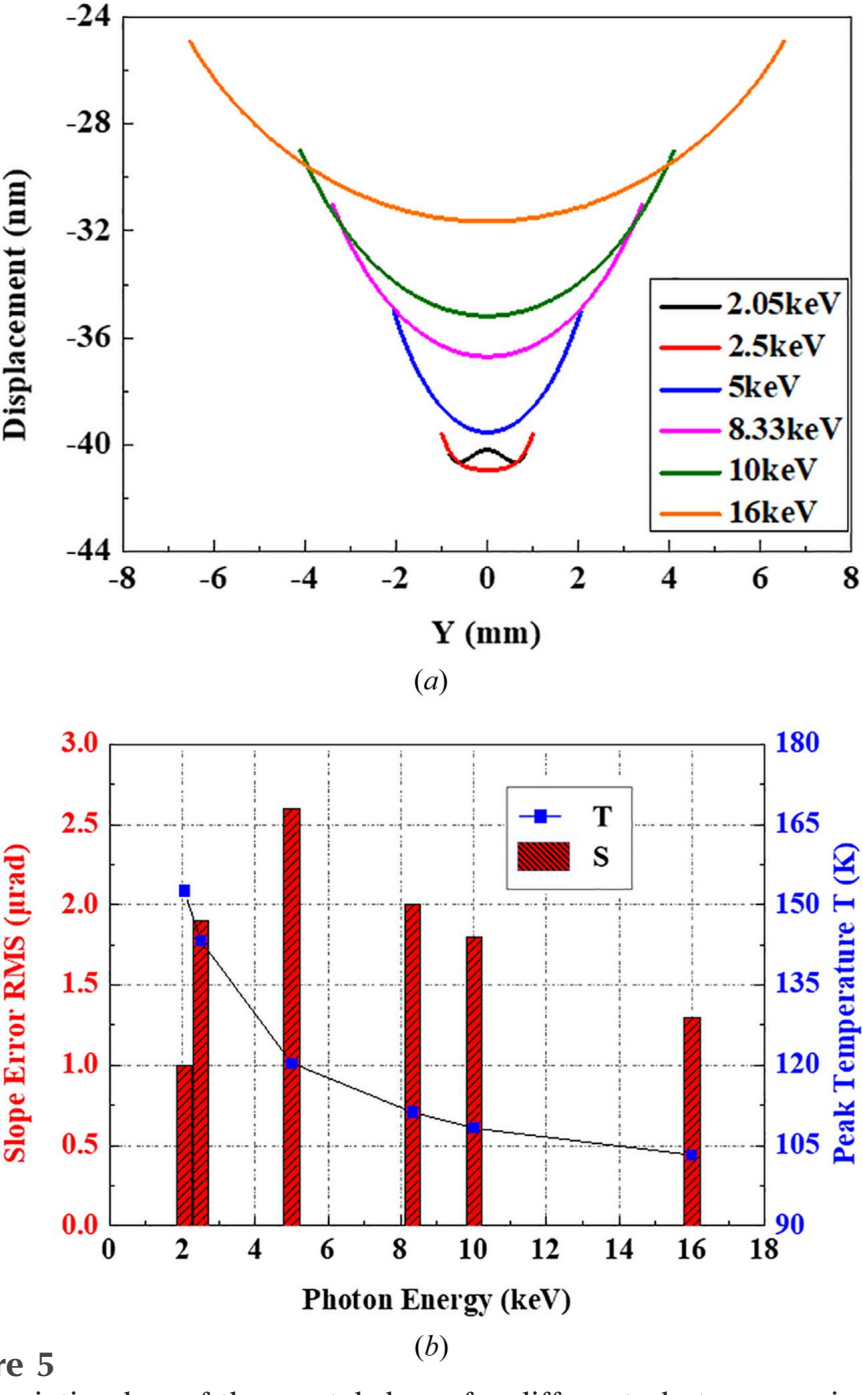
The variation law of the crystal shape for different photon energies with fixed cooling mechanism. (*a*) Displacement of the footprints when CW = 11 mm. (*b*) Variation of slope error RMS and peak temperature by photon energy when CW = 11 mm.

The dedicated monochromator is designed to operate for a photon energy ranging from 2.05 to 16 keV with a pair of Si (111) crystals. We calculate the RMS thermal slope errors of the crystal with an optimal CW of 11 mm at six photon energy positions under the same absorbed heat power as described in Section 2.1. As shown in Fig. 5 ▸(*a*), the crystal surface profile along the footprint length varies from basically flat to concave, then the concave trend gradually decreases and finally tends to flatten again with an increase of photon energy. Note that the beam footprint on the crystal surface becomes longer and the projected power density is reduced by a factor of 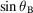 as the photon energy increases. Fig. 5 ▸(*b*) indicates that the thermal slope error can be controlled to less than 3 µrad over the entire photon energy range. The bar graph shows a trend that the slope error firstly increases and then decreases with increasing photon energy, accompanied by a gradually decrease of the crystal peak temperature. The minimum thermal slope error of 1 µrad appears at 2.05 keV as discussed in Section 3.1, while the maximum is 2.6 µrad at 5 keV. The main factor leading to the variation of the profile curve is the positive/negative transformation of the CTE of Si. The optimal peak temperature results in a local flat shape (*i.e.* 152.6 K @ 2.05 keV) as previously stated. In the range 2.05–5 keV, the peak temperature is higher than 125 K, and the coefficient of thermal expansion is positive. With an increase of photon energy, the crystal temperature decreases, the surface centre contracts to a concave shape, and the crystal profile deteriorates. When the temperature decreases to below 125 K (5–16 keV), the CTE turns negative, and the concave surface gradually rebounds; besides, the temperature gradient is also decreasing, thus the crystal profile is improved.

Although all thermal slope errors over the entire photon energy range are less than 2.6 µrad for a CW of 11 mm, the higher-energy beams have worse slope errors, which is obviously unreasonable. In order to achieve more reasonable thermal slope error arrangements over the entire energy range, modifications should be made. In this case, when CW is 11 mm, the heat transfer efficiency is relatively excessive for higher photon energies/large footprints. The higher energy situations are more sensitive to surface shape errors, while the peak temperatures are much lower than their individual optimization points. Then, a compromised design with appropriately reduced CW should be taken in order to obtain the best thermal slope error arrangements over the entire energy range.

We have also further explored the crystal thermal slope errors over the entire operating energy range with various CW. The results indicate that a fixed CW can hardly satisfy all the working conditions. Moreover, there are some deviations for the overall slope error on the crystal, due to, for example, mechanical-induced influences, a different film cooling coefficient from the simulation, and a varied absorbed power due to the accuracy of the primary slits opening, scattering *etc*. in practice. To be most generally applicable, we propose a complementary means for slope error minimization by varying the coolant flow rate appropriately, combined with the PCM.

Generally, the flow rate of LN2 is adjusted with a cryo-cooler in the range 1–10 L min^−1^, and the Reynolds number is between 14000 and 140000, which accords with the turbulence criterion. The relation between the heat transfer efficiency and the flow velocity of the coolant through a circular channel is exhibited by the following equation (Jin *et al.*, 2021 ▸; Dittus & Boelter, 1930 ▸), where typical parameters are shown after in brackets,

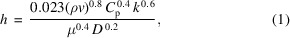

where *D* is the diameter of the coolant (LN2) cooling tube (6 mm); ρ is the density of LN2 (797.8 kg m^−3^); *v* is the flowing velocity of LN2; *C*_p_ is the specific heat capacity of LN2 (2048.2 J kg^−1^ K^−1^); μ is the viscosity of LN2 (0.00015 Pa s); and *k* is the thermal conductivity of LN2 (0.14 W m^−1^ K^−1^).

A closed-loop pressure is usually maintained between 2 and 5 bar, which can ensure the nitro­gen is in a liquid state without vaporization at a temperature no higher than 94 K (*i.e.* upper limit of the cooling channel temperature). The temperature of the cooling channel and the Cu/Si interface (*T*_ambient_) are to some extent affected by the coolant flow rate, under a given heat load and cooling mechanism. For convenience, when a full-length cooling mechanism is chosen, through simulation, the qualitative relationship between the LN2 flow rate and *T*_ambient_ is shown in Table 2 ▸. With an increase in the LN2 flow rate from 1.4 L min^−1^ to 10 L min^−1^, the temperature of the cooling channel wall decreases from 88.95 K to 80 K, and *T*_ambient_ maintains a gap of around 1.6 K higher than that of the cooling channel all the time. Furthermore, as the flow rate of LN2 continues to increase, or by modifying the cooling mechanism, it is reasonable that *T*_ambient_ can be controlled accordingly in the range 80–90 K. For diverse cooling mechanisms, the same value of *T*_ambient_ corresponds to different LN2 flow rates. Thus, the input *T*_ambient_ is changed representing the LN2 flow rate adjustment in the following simulations.

**Figure 6:**
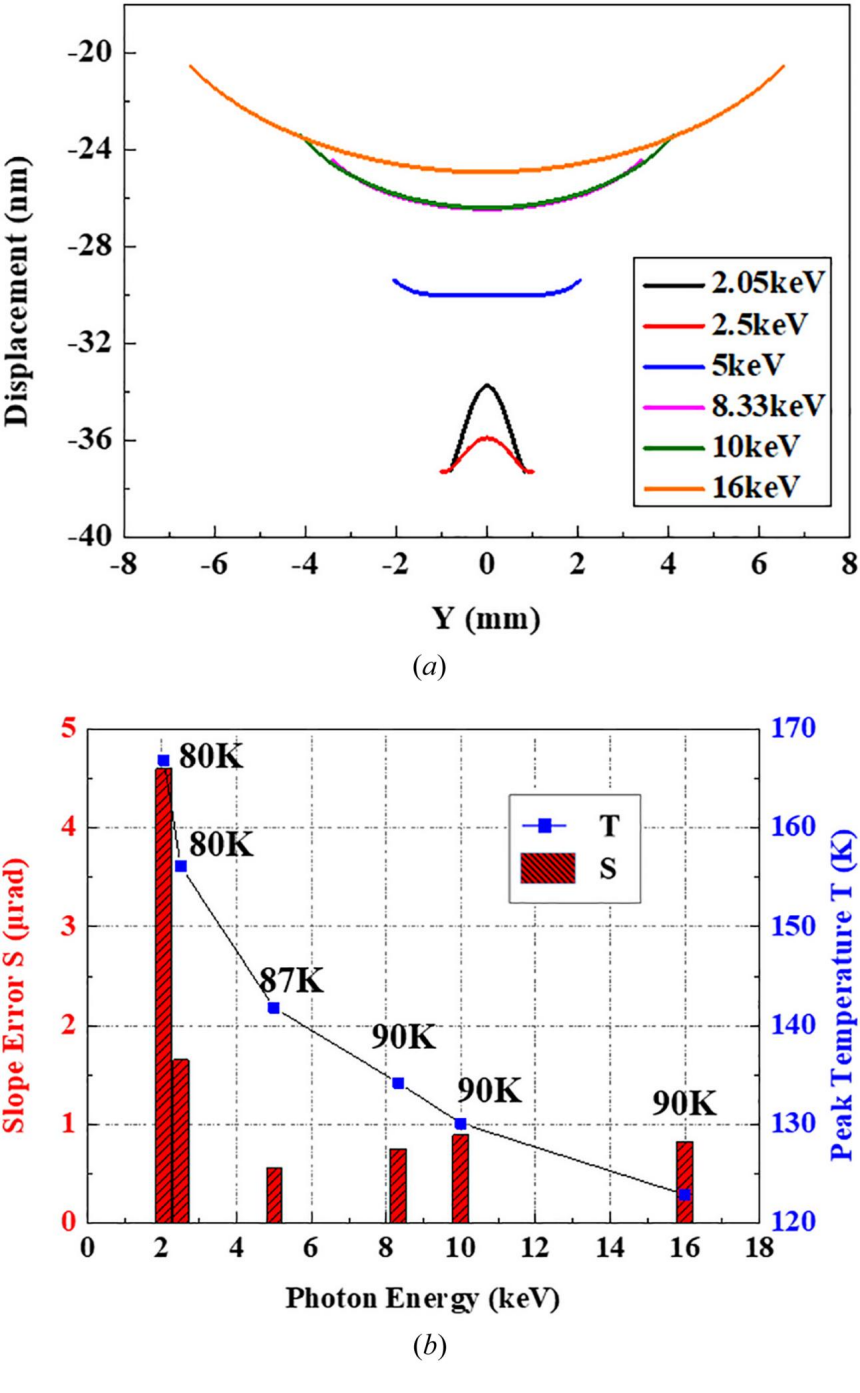
Crystal thermal deformation results for various photon energy conditions with a CW of 9.5 mm and the proper LN2 flow rates. (*a*) Displacement within the footprints. (*b*) Variation of slope error RMS and peak temperature with photon energy and respective optimization *T*_ambient_.

As discussed above, we recalculate the crystal thermal deformation with a new optimal CW of 9.5 mm, combining with the optimized LN2 flow rate (*T*_ambient_ is changed between 80 and 90 K) over the entire photon energy range. The results of thermal deformation and peak temperature are plotted in Fig. 6 ▸. The crystal profile of the footprint area is a convex shape at 2.05 keV and 2.5 keV even though the minimum *T*_ambient_ of 80 K is imposed to achieve a high cooling efficiency, while it is a concave shape from 5 keV to 16 keV as *T*_ambient_ increased from 87 K to 90 K to approach the ‘sweet point’ for the optimized profile. Fig. 6 ▸(*b*) shows that better thermal slope errors within 0.9 µrad can be achieved at higher X-ray energies (5, 8.33, 10 and 16 keV) and below 4.6 µrad at softer energies (2.05, 2.5 keV). Although the thermal slope error becomes larger than that with a CW of 11 mm at low photon energies, it only broadens the intrinsic width by far less than 1%, whose influence is negligible for energy resolution and flux. On the contrary, the thermal slope error is as small as 0.7–0.8 µrad at the common operation energy of 8.33 keV, which is obviously smaller than before, accounting for approximately 3% of the intrinsic width. Therefore, it is necessary to compromise the crystal slope error management over the entire photon energy range during the thermal deformation optimization design of a monochromator.

Thermal deformation can also cause a lensing effect due to wavefront deformation. Lensing effects at low-energy conditions (2.05 keV and 2.5 keV) with relatively large surface shape errors are validated in order to further determine the applicability of the PCM technology over the entire energy range. Ray-tracing simulations are carried out using *SHADOW* code (Rio *et al.*, 2011 ▸). The IVU22 source is adopted as described in Section 2.1. In the beamline layout, a toroidal mirror with a coating stripe of rhodium, as the first optical element, is located at a distance of 31.5 m from the light source, which collimates the X-ray beam in the vertical direction, and simultaneously focuses the beam horizontally to a secondary source at a distance of 15 m downstream. The crystal thermal deformation results by FEA with a CW of 9.5 mm are considered in the ray-tracing process. This is realized by exporting the thermal deformation displacement data from the FEA results, in matrix format, and substituting it into the *SHADOW* software as a surface error of the monochromator. The beam size and angular divergence are captured in the plane perpendicular to the beam direction at the secondary light source (46.5 m from the source).

The ray-tracing results with and without monochromator thermal deformation are shown in Table 3 ▸. At 2.05 keV, the vertical beam size variation owing to thermal deformation is almost negligible, although the vertical divergence has increased by about 11 µrad. At 2.5 keV, the horizontal and vertical beam size are both increased by 3.4%. Nevertheless, these can be ignored as the parameters given by *SHADOW* for the beam size (FWHM) could have a ±5% error bar due to the statistics and fitting algorithm. Therefore, the thermal slope errors of 4.6 µrad and 1.6 µrad have almost no effect on the beam size or divergence at 2.05 and 2.5 keV. In summary, the crystal thermal profile optimized by the PCM can also meet the experimental requirements with a high accuracy over the entire photon energy in this case.

### 3.3. Feasibility under various heat loads

**Figure 7:**
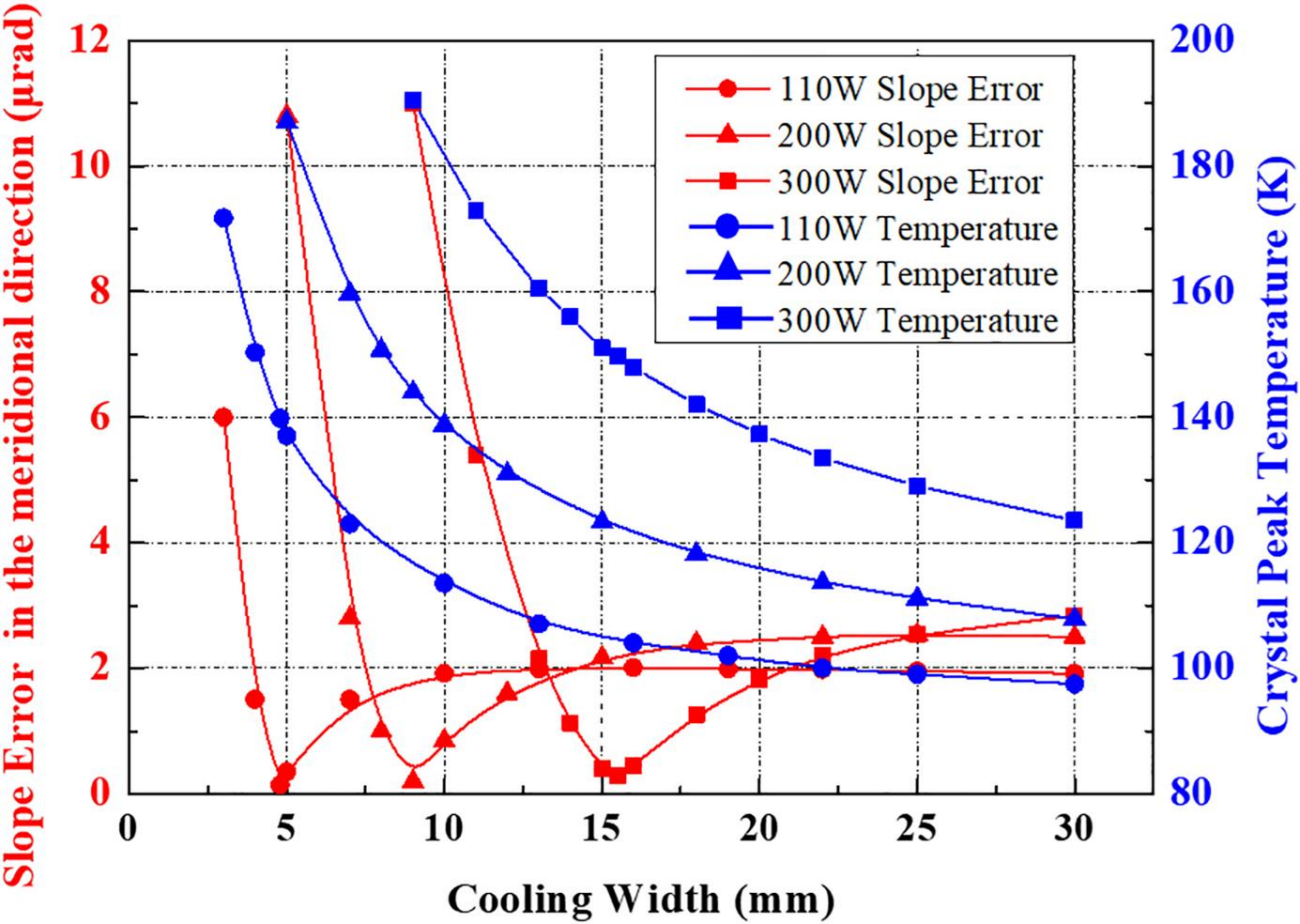
Variation of crystal peak temperatures (blue curves) and thermal slope errors (red curves) with cooling width under different heat power at 8.33 keV energy.

The above cooling scheme optimizations are performed under a total absorption power of 110 W. In order to verify the capacity or feasibility of this cooling model, we perform the crystal thermal deformation calculations again at 8.33 keV under higher heat loads. Herein, three total absorbed heat powers of 110 W, 200 W and 300 W are imposed on the monochromator crystal by modifying the aperture of the primary slits, located at 25 m from the source, corresponding to angular acceptances of 45 µrad × 45 µrad, 60 µrad × 60 µrad and 75 µrad × 75 µrad, respectively. Note that the footprint lengths (in the *Y* direction) are 6.83 mm, 9.1 mm and 11.38 mm, respectively, with the increased angular acceptance. The thermal slope error and crystal peak temperature *versus* the CW under different heat loads are depicted in Fig. 7 ▸.

It can be seen that the variation trends of the thermal-slope-error/CW curve of the crystal under different total absorption powers are consistent with the previous analysis. There always exists an optimal CW with different heat load, that changes depending on the beam size and total power. The higher the thermal load, the larger the cooling width. The crystal peak temperature is in the range 125–165 K as predicted before. The minimum thermal slope error is only 0.14 µrad under an absorbed power of 110 W, achieved at 139.8 K, whereas, for 300 W, the best slope error becomes slightly worse, and can still reach below 0.3 µrad when *T*_p_ is 149.7 K. The optimum CW is 15.5 mm for 300 W, whose upper limit is 30 mm (*i.e.* full-length cooling), indicating that this cooling technology can fully meet demand even for higher power, at 8.33 keV. As revealed previously, the absorbed power is expected to amount to approximately 86–90% of the total power of the incident radiation. Consequently, the upper limit of the heat load imposed on the Si (111) monochromator by PCM optimization should be even higher.

## 4. Conclusions

To achieve a sub-µrad thermal slope error, or even smaller, for advanced cryo-cooled optics under intense heat load, few cooling schemes can control the crystal profile deformation accurately, even by improving the cooling efficiency and decreasing the crystal temperature further. In this paper, a partial cooling method to optimize the thermal deformation of an indirectly cryo-cooled silicon crystal monochromator is proposed and verified. The crystal thermal slope error can be minimized by optimizing the contact cooling area between the crystal and side heat absorbers. In addition, the optimized crystal peak temperature varies as the footprint size changes at different photon energies, which is investigated. A proper cooling mechanism is optimized to fulfil the distortion requirements over the entire photon energy range. A feasible thermal deformation controlling strategy for the cryo-cooled silicon monochromator is suggested in the optical design. In particular, for common working conditions (8.33 keV), the crystal distortion is controlled to as small as 0.3 µrad RMS at an absorption heat power of 300 W with a peak power density of 44.8 W mm^−2^ normal incidence beam by the PCM, demonstrating extremely high thermal load adaptability. The PCM is an effective cooling scheme for high-heat-load monochromators, and provides practical guidance for advanced optics design on DLSRs or FELs.

## Figures and Tables

**Table 1:** Basic thermal-mechanical property parameters used in the simulations (Zhang *et al.*, 2003 ▸, 2014 ▸)

Material	Si			Cu (OFHC)
Density (kg m^−3^)	2329			8900
Orientations	[1, 1, 1]	[1, 1, 0]	[1, 0, 0]	Isotropy
Young’s modulus (GPa)	169	130	130	115
Poisson’s ratio	0.28			0.343
Coefficient of thermal expansion (K^−1^)	Temperature dependent			1.77 × 10^−5^
Thermal conductivity (W m^−1^ K^−1^)	Temperature dependent			391

**Table 2:** Variation of the convective heat transfer coefficient of LN2/Cu, the temperature of the cooling channel wall and the Cu/Si interface temperature with the flow rate of LN2

Flow rate of LN2 (L min^−1^)	Film coefficient of LN2/Cu (W m^−2^ K^−1^)	Temperature of cooling channel wall (K)	Temperature of Cu/Si interface (K)
1.4	3000	88.95	90.3
2.03	4000	88.4	90
2.7	5000	86.1	87.7
3.4	6000	84.6	86.2
5	8000	82.7	84.2
6	10000	81.6	83.1
10	15000	80.0	81.6

**Table 3:** Ray-tracing results of the X-ray beam at the secondary source

		Without thermal deformation		With thermal deformation	
Photon energy (keV)	Thermal slope error (µrad, RMS)	Beam size (µm)	Divergence (µrad)	Beam size (µm)	Divergence (µrad)
2.05	4.6	29.1 × 574.1	39.4 × 0.67	29.6 × 570.0	40.0 × 11.8
2.5	1.6	29.2 × 493.2	34.3 × 0.63	30.2 × 510.0	36.2 × 12.4
